# Boron Nitride Nanotube (BNNT) Membranes for Energy and Environmental Applications

**DOI:** 10.3390/membranes10120430

**Published:** 2020-12-16

**Authors:** Numan Yanar, Eunmok Yang, Hosik Park, Moon Son, Heechul Choi

**Affiliations:** 1 School of Earth Sciences and Environmental Engineering, Gwangju Institute of Science and Technology (GIST), 123-Cheomdangwagi-ro, Buk-gu, Gwangju 61005, Korea; numanyanar@gm.gist.ac.kr (N.Y.); yang1990@gist.ac.kr (E.Y.); 2 Green Carbon Research Center, Chemical Process Division, Korea Research Institute of Chemical Technology (KRICT), Daejeon 34114, Korea; hspark@krict.re.kr; 3 School of Urban and Environmental Engineering, Ulsan National Institute of Science and Technology, 50, UNIST-gil, Eonyang-eup, Ulju-gun, Ulsan 44919, Korea

**Keywords:** boron nitride nanotubes, BNNT membranes, water filtration, gas separation, thermal membranes, battery separators

## Abstract

Owing to their extraordinary thermal, mechanical, optical, and electrical properties, boron nitride nanotubes (BNNTs) have been attracting considerable attention in various scientific fields, making it more promising as a nanomaterial compared to other nanotubes. Recent studies reported that BNNTs exhibit better properties than carbon nanotubes, which have been extensively investigated for most environment-energy applications. Irrespective of its chirality, BNNT is a constant wide-bandgap insulator, exhibiting thermal oxidation resistance, piezoelectric properties, high hydrogen adsorption, ultraviolet luminescence, cytocompatibility, and stability. These unique properties of BNNT render it an exceptional material for separation applications, e.g., membranes. Recent studies reported that water filtration, gas separation, sensing, and battery separator membranes can considerably benefit from these properties. That is, flux, rejection, anti-fouling, sensing, structural, thermal, electrical, and optical properties of membranes can be enhanced by the contribution of BNNTs. Thus far, a majority of studies have focused on molecular simulation. Hence, the requirement of an extensive review has emerged. In this perspective article, advanced properties of BNNTs are analyzed, followed by a discussion on the advantages of these properties for membrane science with an overview of the current literature. We hope to provide insights into BNNT materials and accelerate research for environment-energy applications.

## 1. Introduction

Owing to their exclusive and attractive physical, chemical, and mechanical properties, nanomaterials have been intensively used in various scientific fields to overcome the current limitations of conventional materials [1]. With dimensions of less than 100 nm, nanomaterials exhibit extremely unique properties, including a high surface-to-volume ratio, high catalytic properties, unique surface charges, and chemical composition properties [2]. Therefore, nanomaterials are typically used in various fields of science and engineering, including pharmaceuticals, the electronic industry, energy applications, the biomedical sector, wear and corrosive-resistant coatings, and physical separation. Among these areas, physical separation, especially membrane science, has extensively adopted nanomaterials for polymeric and inorganic membranes either by embedding nanoparticles into porous membranes or by simple blending during fabrication [3,4]. Metal-based nanoparticles such as metal oxides and metal-organic frameworks (MOFs), silica-based nanoparticles, biomaterials, and carbon derivatives (e.g., carbon nanotubes (CNTs), carbon quantum dots, graphite, and graphene) are frequently used nanomaterials for membrane fabrication as surface modifiers or blending materials in the polymeric membrane matrix [5,6]. Recently, boron nitride nanotubes (BNNTs) have been attracting tremendous attention due to their unique properties, including high permselectivity, mechanical strength, thermal conductivity, electrically insulating behavior, piezoelectric properties, neutron shielding capability, oxidation resistance, etc. [7].

Since the invention of BNNTs in 1995 by Chopra et al. [8], several achievements enabling access to this material as well as promising applications have been reported [7]. Recently, BNNTs have also been investigated for membrane fabrication. Although few key studies exist, most of them are still in the early or preliminary stage. Therefore, the requirement of an extensive perspective article has emerged to focus the attention of researchers on this novel membrane material. As an effort toward providing the first review on this topic, we hope to provide insights into BNNT materials and accelerate research in the field of membranes for environment-energy applications. 

## 2. Boron Nitride Nanotubes (BNNTs)

Layered boron nitride (BN) is structurally similar to graphite, in which boron (B) and nitrogen (N) atoms are replaced by carbon (C) atoms. Therefore, BNNTs resemble a rolled graphite-like BN sheet, comprising N and B atoms instead of C atoms [9]. BNNTs can be classified on the basis of their walls, longitudinal growth, and chirality.

According to their walls, BNNTs can be classified as single-walled BNNTS (SBNNTS) and multiwalled BNNTs (MBNNTs) even though SBNNTS have been reported to a relatively lesser extent in research and applications [10]. Different from the covalent C–C bonds found in CNTs and graphene, ionic interactions exist between neighboring BN layers as a result of the partial ionic character of the B–N bond [9].

**Figure 1:**
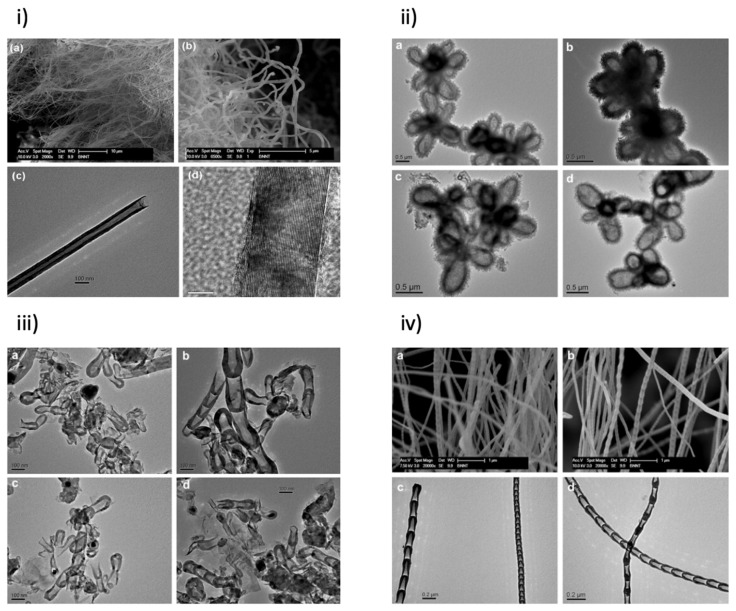
SEM and TEM images of (**i**) straight-walled, (**ii**) flower-type, (**iii**) short-bamboo-type and (**iv**) long-bamboo-type BNNTs (Reproduced from a previous study of [11] published by Elsevier).

According to the longitudinal growth of BNs, BNNTs can be classified as straight-walled, flower-type, short bamboo-type, and long bamboo-type BNNTs. By applying different temperatures (1000 °C to 1150 °C) and maintaining the NH_3_ gas flow constant at 50 sccm, Reddy et al. [11] synthesized flower-type BN nanostructures (1000 °C), short bamboo-type BN nanostructures (1050 °C), long bamboo-type BNNTs (1100 °C), and straight-walled BNNTs (1150 °C) (Figure 1) [11].

**Figure 2:**
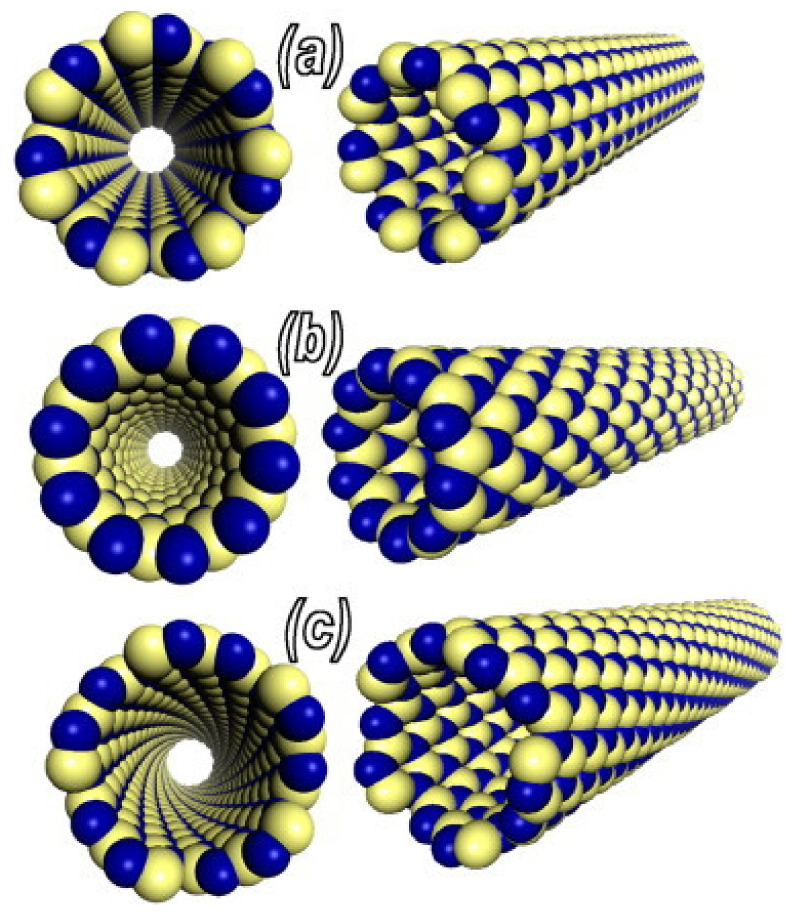
Atomic models of (**a**) armchair; (**b**) zigzag; (**c**) chiral BNNTs. (Reproduced from a previous study of Zhi et al. [15]).

In addition, BNNTs are classified according to their chirality. BNNTs comprise honeycomb BNs (h-BNs) that are connected from two lattice points. These two points coincide after the strip is rolled up, affording a seamless cylinder so that the two points coincide. The lattice translation vector between the two points decides the radius and chiral angle (θ) of the tube. The resultant tubes are classified as achiral for θ = 0 (zigzag) and θ = π/6 (armchair) and chiral for 0 < θ < π/6 [12,13,14] (Figure 2).

### 2.1. BNNT Synthesis Methods

The first synthesis of BNNT [8] was conducted in a plasma arc discharge apparatus that was previously used to fabricate carbon fullerene [16]. After the first synthesis of BNNT, ball milling, annealing methods, chemical vapor deposition (CVD) using borazine [17], induction heating of boron oxide CVD (BOCVD), continuous laser ablation, BN substitution method from CNT templates, plasma-enhanced pulsed laser deposition and the catalyst-free pressurized vapor/condenser method were employed for the efficient production of BNNTs [9,12,18,19]. Furthermore, induction-coupled plasma synthesis [20], direct induction [21] and boron ink annealing [22,23] methods also have been employed to fabricate BNNTs. Table 1 lists the advantages and disadvantages of different fabrication methods for BNNTS. 

### 2.2. Composites of BNNTs

Although a majority of composite studies reported the fabrication of polymer and ceramic composites, a few studies have also reported metal composites. As a composite material, BNNTs render high thermal conductivity, optical properties, mechanical strength, wettability, electrical resistivity, and antioxidation abilities to the composite polymer, ceramic, or metal [37], as well as being the strongest lightweight nanomaterial with a Young’s modulus of ~1.2 TPa [38]. These extraordinary properties of BNNTs also make them an alternative to CNTs. For several composite applications of CNTs, BNNTs exhibit better properties. The comparison of BNNT and CNTs (Table 2) clearly revealed that BNNTs are a better option for composites where electric insulation or high thermal properties are required. 

Owing to their pure white/transparent color and high mechanical strength, BNNTs render immense benefits to polymer composites for further applications. Therefore, BNNT polymer composites demonstrate considerable promise for applications in various scientific fields. However, a key disadvantage of BNNTs is that they cannot disperse easily in water and in most organic solvents. That being said, better dispersion properties can be obtained by functionalization. Functionalized BNNTs further provide better interfacial interactions in composite materials, better molecule loading ability, biological application exploration, etc. [55]. The first fabrication of a polymer composite of BNNTs was carried out through functionalization based on interactions between the amino functional groups of polyethylene glycol (PEG) molecules and the nanotube surface of borons [56]. Later, BNNTs wrapped with poly[m-phenylenevinylene-co-(2,5-dioctoxy-p-phenylenevinylene)] (PmPV) were investigated, which were completely soluble in chloroform, N,N-dimethylacetamide, and tetrahydrofuran, etc. [57]. Currently, various polymers can be used for the functionalization of BNNTs.

The structural and mechanical durability of BNNTs under high-temperature environments [58] makes them ideal materials for the fabrication of metal and ceramic composites that require high processing temperatures. Furthermore, the thermal conductivity of ceramic composites reinforced with BNNTs was reported to be improved, which was found to be related to the high thermal conductivity (200 W/(m·K)) of BNNTs [59,60]. Therefore, currently, studies have focused on BNNT-Al alloys and ceramic or glass composites of BNNTs, suggesting that BNNTs are highly effective at enhancing mechanical and thermal properties of their metal and ceramic composites [61].

### 2.3. BNNT Applications

BNNTs exhibit extremely unique properties; hence, they are a promising material for various applications. As discussed above, currently employed fabrication methods still exhibit some issues that need to be addressed. By the introduction of new fabrication methods, BNNTs are currently commercially available, even though they are still in the early stage of development. BNNTs were first commercialized in 2014 at the invention of the National Aeronautics and Space Administration (NASA) Langley Research Center [21,36], which has widened their applications. In addition, commercial BNNTs exhibit the advantages of their nanoscale size and high aspect ratio similar to those of CNTs. However, they are advantageous when the functional properties of CNTs need to be preferentially avoided. Furthermore, BNNTs exhibit better properties in terms of wetting, mechanical strength, thermal resistance, and electrical insulation properties. Electronic, drug delivery [62], nano-medicine [63], biomedical [64], aerospace [65], energy [66], and environmental applications can considerably benefit from these properties. In particular, composites of BNNTs can be used for the enhancement of mechanical reinforcement and structural composites as well as of thermal conductivity, high-temperature material processing, high-temperature applications (thermal barriers, fire resistance, etc.), electrical insulation, piezoelectric sensing, energy harvesting, neutron shielding, and transparency of composites [61]. These wide application ranges of BNNT composites are further important for membrane science. Currently, various studies focusing on polymer or ceramic membranes derive benefits from BNNTs.

## 3. BNNT Membranes

Nanotubes have been attracting considerable attention as membrane materials due to their high surface-to-volume ratio. Various studies on membranes employing CNTs [67], halloysite nanotubes (HNTs) [68,69], silicon nanotubes [70], TiO_2_ nanotubes [71,72], and BNNTs [73] are available. In particular, CNT composites demonstrate high performance in terms of permeability, selectivity, fouling/biofouling, mechanical strength, and thermal properties for membrane applications [67]. However, as an alternative, BNNTs have recently been introduced to membrane science. As BNNTs exhibit properties similar to those of CNTs, a majority of the applications of CNTs are also applicable to BNNTs. In fact, BNNTs exhibit better properties in terms of wettability and water permeation, thermal conductivity and resistance, and mechanical strength when they are used as a composite material for membranes.

### 3.1. Wettability and Water Permeation

Membrane wettability is a key parameter for water or vapor permeation properties. Hydrophilic membranes are mostly preferred for high water permeation. For several years, various nanomaterials have been used as composite materials to enhance surface hydrophilicity, which is an extremely crucial factor for water permeation. Functionalized CNTs [74,75], HNTs [68,69,76], graphene derivatives [77,78,79,80], metal oxides [81,82], cellulosic materials [83,84], and bio-materials (e.g., aquaporin, amphiphilics, or lignin) [85] are the most commonly used nanomaterials. Nevertheless, hydrophobicity is also important depending on the type of membrane. Various nanomaterial applications for enhancing the hydrophobicity of membranes are available, including distillation, pervaporation, gas separation, gas adsorption, and oil–water separation membranes [86]. Compared to other nanomaterials, BNNTs exhibit unique wettability properties, which are tunable. BNNT films exhibit high hydrophobic properties with contact angles of greater than 170° [87]; however, they can be tuned to the desired wettability, even to superhydrophilicity (CA < 5°), by using N_2_/H_2_ gas plasma of different energy inputs and modes [51] or using a combination of pulsed and continuous wave mode plasma [88].

Water permeation can also be further enhanced through sub-nanometer water channels of BNNTs. Won et al. reported with a theoretical study that water molecules can permeate through (5,5) BNNT. The hydrogen bonding and axial diffusion coefficient of the water inside the (5,5) BNNT sub-channel were only comparable to those of higher-diameter (6,6) CNT (1.3 Å greater than the diameter of (5,5) BNNT), while CNT with a similar diameter was only capable of filling water rather than permeation [89]. By considering this property of BNNTs, the vertical alignment of BNNTs becomes extremely crucial especially for the fabrication of ion-selective membranes. 

### 3.2. Thermal Conductivity and Resistance

Thermal conductivity without oxidation is an extremely difficult property to obtain for membranes. Jia et al. reported that BNNT polymer composites can withstand temperatures of up to 900 °C in an oxidizing environment [60]. Zhi et al. reported that BNNT composites of poly(methyl methacrylate) (PMMA), polystyrene (PS), poly(vinyl butyral) (PVB), and poly(ethylene vinyl alcohol) (PEVA) exhibit 21.1 (PMMA), 20.1 (PS), 7.5 (PVB), and 14.7 (PEVA) times better thermal conductivity than those of the pure polymers [90]. This property is important for high-temperature membrane separation, such as in the case of proton exchange membranes (PEMs) [91]. Furthermore, the high thermal conductivity of BNNT composites, which can increase the thermal conductivity in a ratio of greater than 2000% [60], would further be beneficial for thermal energy recovery by using thermal separation membranes.

### 3.3. Mechanical Strength

In most of the applications in which BNNT nanomaterials have been used as composites, the mechanical strength is efficiently enhanced as long as the materials are not overused, which may lead to inductility or brittleness due to agglomeration or low adhesion [92]. BNNTs can considerably enhance the mechanical strength of its glass [93], ceramic [94], polymer [48], and metal composites [95]. Previously, compared to CNTs, BNNTs were reported to form much stronger binding interfaces with polymers [48]. This result is also further related to their mechanical strength as the low diameter of BNNTs leads to high adhesion, which can increase the mechanical strength of its composites. 

### 3.4. Electrical Properties

Due to the similarities between BNNTs and CNTs, the other properties of BNNTs were mostly discussed in comparison with CNTs. However, in terms of electrical insulation, BNNTs exhibit properties extremely different from those of CNTs. BNNTs are electrically insulating materials, whereas CNTs are conductive materials. Unlike delocalized π electrons in CNTs, π electrons are localized in BNNTs, rendering electrical insulation [37,96]. Electron insulation properties of BNNTs can immensely benefit battery applications for separator membranes. This will be further discussed in subsequent sections.

Kim et al. discussed another important electrical property of BNNTs [96]. BNNTs exhibit a band gap between 5 and 6 eV [7]. However, Kim et al. reported that the band gap of zigzag BNNTs can be tuned under high mechanical pressure. Radial deformations under transverse pressures of about 10 GPa can lead to the reduction of band gap to 1 eV via the collapse of the BNNTs down to the van der Waals interlayer spacing [96]. Furthermore, covalent functionalization is also effective at reducing the band gap of BNNTs [97]. These studies revealed that BNNTs also can exhibit metallic behavior with a tunable bandgap.

BNNTs are also nonpolar piezoelectric materials, which exhibit a strain response greater than that of polar polymers [98]. This property should be further considered for biofouling studies of membranes.

## 4. Perspectives on Key Membrane Studies

### 4.1. Water Treatment Membranes

**Figure 3:**
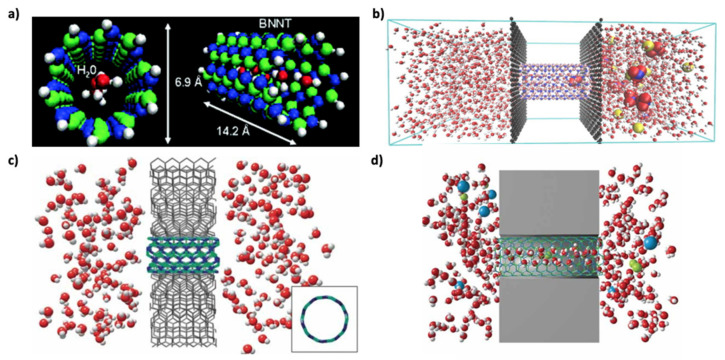
Molecular dynamic simulations of water filtration through BNNTs: (**a**) Effective permeation of water molecules through (5,5) BNNT (Reprinted (adapted) with permission from [89]. Copyright (2020) American Chemical Society); (**b**) Water transport and nitrate ion selectivity simulation of a BNNT embedded between two graphene sheets as membranes (Reproduced from a previous study [100] with the permission of Elsevier); (**c**) Water molecule transport with a 100% salt rejection through the (5,5) BNNT-embedded silicon-nitride membrane (Reproduced from a previous study [101] with the permission of Wiley); (**d**) Anion–cation-selective BNNT-embedded silicon-nitride membrane (Reproduced from a previous study [102] with the permission of Wiley).

**Figure 4:**
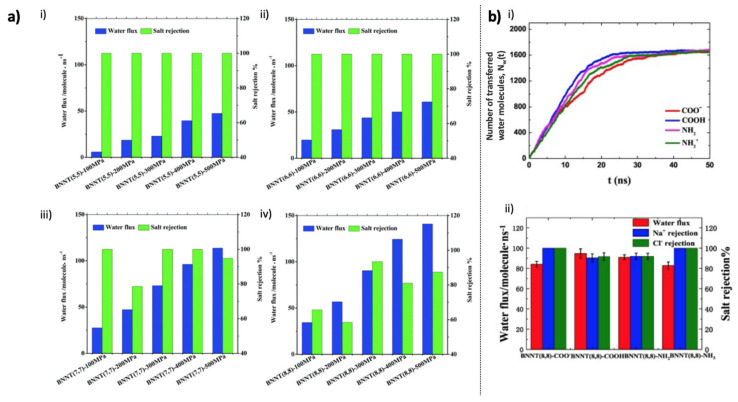
Water flux and salt rejection performances of BNNT-embedded desalination membranes: (**a**) Membranes with different BNNT diameters (Reproduced from Ref. [105] with permission from The Royal Society of Chemistry); (**b**) Membranes with functionalized BNNT (8,8)-COO^−^ and BNNT (8,8)-NH_3_^+^ (Reproduced from a previous study [106] with the permission of Elsevier).

Even though Won et al. reported a water transport comparison study between BNNT and CNT [89] (Figure 3a), which is discussed above, Suk et al. reported the first membrane study of BNNTs via a molecular dynamics (MD) modeling approach of reverse osmosis transportation of water molecules through a CNT, a BNNT, and a single pore of PMMA [99]. Non-equilibrium MD simulation revealed that the water flux performance of BNNT is slightly greater than that of CNT and significantly greater than that of the PMMA pore [99]. Later, Azamat et al. performed water transport and nitrate ion selectivity simulation of BNNTs with different diameters (including (4,4), (5,5), (6,6), (7,7), (8,8), and (9,9) BNNTs) embedded between two graphene sheets as membranes (Figure 3b): The (4,4), (5,5), and (6,6) BNNTs did not permit the passage of nitrate ion, while (4,4) BNNT also did not allow water permeation [100]. Won et al. [89] reported that (5,5) CNTs do not permit the passage of water molecules, but permit their encapsulation, and that BNNTs demonstrated more promise, especially for composite membranes with vertically aligned BNNTs. This point was further investigated by Hilder et al. [101], and MD simulations revealed that (5,5) BNNT embedded in a silicon nitride membrane (Figure 3c) can exhibit a high salt rejection performance of 100%, with the water permeation of 10.7 water molecules per nanosecond (or 0.9268 L m^−2^ h^−1^) [101]. The same research group also reported that the diameter of BNT is effective at separating anions and cations (Figure 3d) [102]. In this regard, Azamat et al. investigated the heavy metal selectivity performance of BNNTs by using Zn^2+^ as a model heavy metal. In the model, (7,7) BNNT- and (8,8) BNNT-embedded silicon-nitride membranes were immersed in a ZnCl_2_ solution. (7,7) BNNT selectively conducted Zn^2+^, while (8,8) BNNT was successful in selectively conducting Cl^−^ [103]. Furthermore, in another study, (7,7) BNNT-embedded silicon-nitride membranes were also effective in removing Cd^2+^ from wastewater [104]. Liang et al. reported that (7,7) BNNT exhibits a better desalination performance efficiency [105]. A remarkably high selectivity ratio of 3071:1 for water molecules over Na^+^ can be achieved through the free-energy barrier gap of Na^+^ and a water molecule passing through (7,7) BNNT (Figure 4a). Simulation results revealed that a 10-cm^2^ nanotube membrane with 1.5 × 10^13^ pores per cm^2^ can permeate freshwater at a flow rate of 98 L d^−1^ MPa^−1^ under a pressure 100 MPa [105]. However, notably, real seawater comprises ions other than Na^+^ that need to be purified. Therefore, polyatomic ions such as nitrate should also be considered. As reported by Azamat et al., (7,7) BNNT is not as efficient for the filtration of nitrate compared to (4,4), (5,5), and (6,6) BNNTs [100]. Zhang et al. simulated the functionalized forms of BNNTs: BNNT (8,8)-COO^−^ and BNNT (8,8)-NH_3_^+^ [106]. The water flux of aligned BNNT (8,8)-NH_3_^+^ was as high as 40 L·cm^−2^·day^−1^·MPa^−1^, while the salt rejection also reached 100% due to the space-steric effect and electrostatic interaction, even though BNNT (8,8)-COO^−^ exhibited a relatively low performance (Figure 4b) [106].

**Figure 5:**
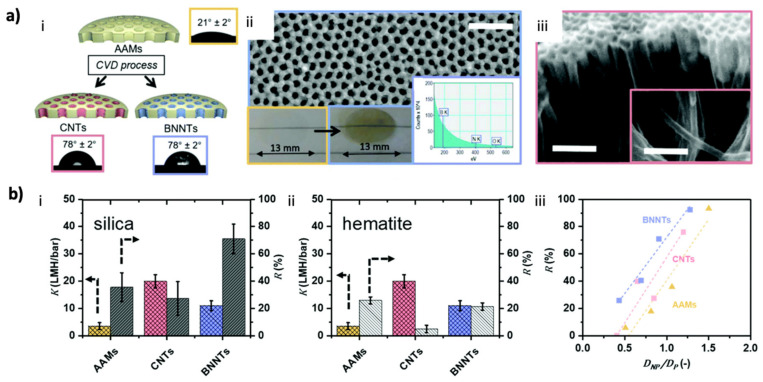
Performance of aligned BNNTs by fabricated membranes and comparison with aligned CNTs ([73] Published by The Royal Society of Chemistry): (**a**) Schematic of the deposition of CNTs and BNNTs in an anodic alumina membrane (AAM): (**i**). optical micrograph of a water droplet and the associated contact angle for each material, (**ii**). FESEM image of BN deposition (before and after) and electron energy-loss spectroscopy, (**iii**). nanotubes released from a cracked membrane after CVD; (**b**) Experimental permeance K and rejection R for (**i**). negatively charged silica (pH = 5.5) and (**ii**). positively charged hematite (pH = 5.3). (**iii**). Calculated silica NP rejection of AAM, CNT, and BNNT membranes as a function of D_NP_/D_P_.

**Figure 6:**
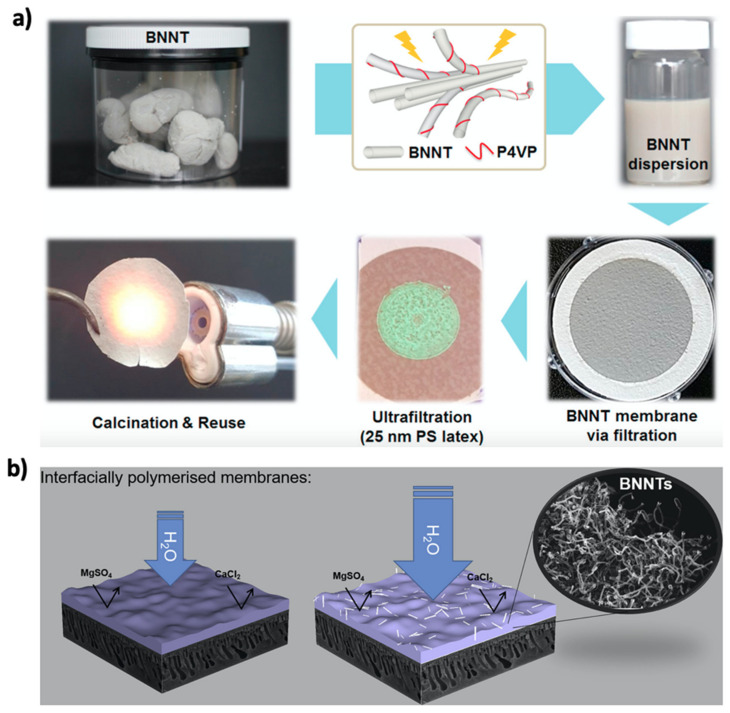
Polymer composite applications of well-dispersed BNNTs: (**a**) fabrication of flame-resistive BNNT ultrafiltration membranes by using a polymeric dispersing agent (P4VP) (Reproduced from a previous study [107] with the permission of Elsevier); (**b**) Thin-film nanocomposite nanofiltration membranes with a BNNT-dispersed polyamide active layer (Reproduced from a previous study [108] with the permission of Elsevier).

The high performance of aligned BNNTs revealed through MD simulations was also supported by in situ laboratory experiments. Casanova et al. compared the performances of aligned BNNT and CNT membranes by using nanotubes with comparable diameters: BNNT membranes exhibited ~40% higher rejection than that of CNT membranes, indicating that a 70% higher permeance can be obtained for a BNNT with a 30% larger diameter while simultaneously maintaining the same rejection rates for CNT and BNNT membranes (Figure 5) [73]. As one of the biggest advantages of BNNT is its flame resistance, Lim et al. introduced regenerable ultrafiltration membranes fabricated by the filtration of thermally stable and highly dispersed BNNTs (Figure 6a) by using poly(4-vinylpyridine) (P4VP) as the BNNT stabilizer with its strong adsorption energy. Even though BNNTs are highly hydrophobic, the BNNT membrane prepared by the P4VP-assisted dispersion exhibited a permeation of 230 L m^−2^ h^−1^ with a >99% removal efficiency of polystyrene and gold nanoparticles [107]. Casanova et al. further reported the application of thin-film nanocomposite membranes of BNNTs by incorporating the nanotubes into a polyamide (PA) active layer via interfacial polymerization on a polyethersulfone support (Figure 6b). A BNNT loading of greater than 0.02 wt% led to agglomeration with the overall loss of performance. Membranes with 0.02 wt% BNNTs in the PA layer exhibited a permeance of 4.5 L m^−2^ h^−1^/bar with >90% rejection of MgSO_4_ and >80% rejection of CaCl_2_. Furthermore, humic acid fouling tests of BNNT membranes revealed a 95% flux recovery ratio and a ~50% lower flux loss than those for membranes without BNNTs. However, membranes with a BNNT loading of greater than 0.02 wt% exhibited performance loss due to nanotube agglomeration [108]. This research is crucial to understand the importance of BNNT dispersion. As similar phenomena were previously noted for composite CNT membranes [109], dispersed BNNT membranes exhibited better performance due to an enhanced nanomaterial contact surface. In this regard, Fernandez-Yague et al. reported the effect of polydopamine functionalization on the good dispersion of BNNTs, which can be further considered for membrane applications [46].

In addition, osmotic energy harvesting applications of BNNTs were investigated. Siria et al. fabricated a hierarchical nanofluidic device comprising a BNNT piercing an ultrathin membrane and connecting two fluid reservoirs. This transmembrane geometry afforded a power density per unit tube surface of ≈4 kW m^−2^ for a single BNNT [110]; this value is vastly greater than the power densities reported for current pressure-retarded osmosis (PRO) membranes [111]. This result suggested that BNNTs can be used as membranes for high-efficiency osmotic power harvesting under a salinity gradient. Furthermore, Cetindag et al. [112] also reported high-osmotic-energy conversion (≈7.5 kW m^−2^ at pH 11 for a 1 M:1 mM KCl molarity difference) through a single BNNT. These studies are paving the way for the full-scale application of PRO membranes.

Current studies have reported that BNNTs are promising materials for water filtration membranes with high permselectivity. However, other issues still need to be addressed for their use as membrane materials. One of the most important issues is membrane fouling. Although the organic fouling performance of BNNT membranes was investigated by the filtration of a humic acid solution, other organic foulants, colloidal foulants, scalants, and bio-foulants should also be considered. In addition, as most studies have reported MD simulations, additional studies that employ in situ filtration for various membrane systems, such as reverse osmosis and forward osmosis/PRO, are required. Moreover, the thermal performance of BNNTs can be further benefited by employing thermal membrane systems such as for membrane distillation and pervaporation. By also considering the advantages and the recent boost in 3D printed membranes [113,114,115,116], BNNTs should also be considered for that field.

### 4.2. Gas. Separation and Sensor Membranes

**Figure 7:**
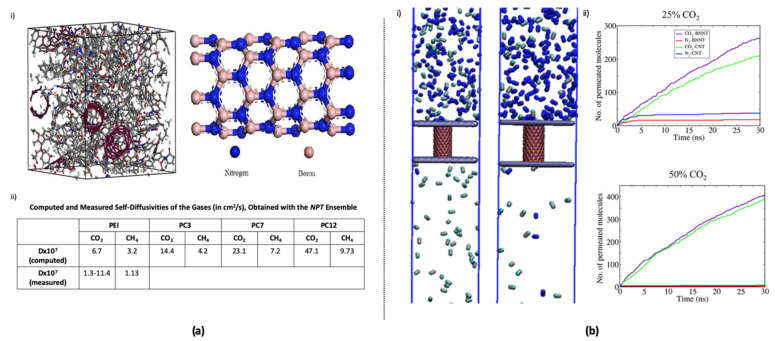
Gas permeation through BNNT membranes: (**a**) PEI-BNNT composite for gas diffusion (Reprinted (adapted) with permission from [117]. Copyright (2020) American Chemical Society) (**i**). 3D image of PEI and three BNNTs in its matrix, (**ii**). self-diffusivities of the gases through pure PEI, PC3, PC7, and PC12; (**b**) Separation of CO_2_ and N_2_ from flue gas using CNT and BNNT membranes (Reprinted (adapted) with permission from [118]. Copyright (2020) American Chemical Society) (**i**). gas permeation through the (14,0) BNNT membrane from a binary mixture of CO_2_ and N_2_ with 50% and 25% CO_2_ concentrations, respectively from left to right, (**ii**). Permeation of gas molecules through (14,0) BNNT membranes from a binary mixture of CO_2_ and N_2_ with 25% and 50% CO_2_ concentrations, respectively, from the top to bottom, at an initial pressure of 50 bar and at 303 K.

Regarding these types of membranes, BNNTs can exhibit additional advantages due to their high mechanical properties. However, only a few studies are available. Wang et al. fabricated amorphous poly(ether imide) (PEI) and composite PEI membranes of zigzag (3,0), (7,0), and (12,0) BNNTs, namely PC3, PC7, and PC12 and investigated them by MD simulations. In addition, solubilities and self-diffusivities of CO_2_ and CH_4_ in the PEI and its composites with BNNTs were investigated. The (3,0) BNNT composite exhibited the highest distributed free volume. Thus, it also exhibited the highest diffusivities of CO_2_ and CH_4_ (Figure 7a). Furthermore, it also exhibited the highest solubility [117]. Maurya et al. conducted an MD simulation for the separation of CO_2_ from flue gas by using CNT and BNNT membranes. The gas separation performance of CNT and BNNT membranes with chiralities of (10,0), (14,0), and (18,0) was investigated using flue gas, which was considered as a binary mixture of CO_2_ and N_2_ with CO_2_ molar concentrations of 25% and 50%. CO_2_ permeance over the BNNT membrane was greater than that over the CNT membrane. (14,0) BNNTs exhibited optimum gas permeance and selectivity, which was better than that of the CNT membrane with the same chirality. The performance of the (14,0) BNNT membrane was better than that of the (14,0) CNT membrane. Although the free-energy change revealed that the permeation of CO_2_ through CNT is more desirable than that through BNNT, the N_2_ permeation resistance of CNT is less than that of BNNT, which can reduce the overall permeation of CO_2_ through the CNT membrane to a greater extent than that through the BNNT membrane (Figure 7b). Therefore, compared to the CNT membrane, the BNNT membrane exhibits higher permselectivity of CO_2_/N_2_ [118]. 

Different from gas separation, a micro-electro-mechanical system (MEMS)-based field effect transistor (FET) sensor for hydrogen detection was reported by Yoo et al. via the modification of the gate electrode with a BNNT-decorated Pd-ternary alloy (Pd_63·2_Ni_34·3_Co_2.5_) as the hydrogen sensing membrane layer. The BNNT-decorated Pd ternary alloy exhibited a high sensing response, fast response, and recovery time for H_2_; low power consumption; long-term stability; and a wide detection range from 1 to 5000 ppm H_2_ [119].

### 4.3. Battery Separator and Proton Exchange Membranes 

Pristine BNNT is known to be a thermo-conductive, but electrically insulating, material, as a result of the constant and wide bandgap of ~5.5 eV for BN [90]. An appropriate combination of its unique electrical, thermal, and structural properties can be exploited in applications where a thin insulator is required, such as in battery research. Rahman et al. introduced a new BNNT-coated PP separator with a thermal stability of up to 150 °C for the safer operation of lithium-ion battery cells [120]. Kentaro et al. suggested that BNNTs can successfully replace a complexed-polymer-based separation compartment in the battery to render a lighter and safer battery. Insulative BNNTs were used as the separator under conductive CNTs, which served as the collector. The electrode/separator stack without the use of any organic polymer or metallic foil stack led to an increase in the active material content to 93.6% with a high thermal resistance at 500 °C [121]. These studies employed nonfunctionalized BNNTs. In this regard, Fan et al. previously addressed the critical issue of polysulfide (PS) leak into the electrolyte in lithium–sulfur (Li–S) batteries. The Li–S cell was coated with functionalized BN sheets (FBN)-graphene composite, and the performance of FBN was compared with that of positively charged amino groups, commercial BN sheets (CBN), and porous BN (PBN). The results revealed that CBN, with a small surface area and unfunctionalized surfaces, cannot adsorb PS due to its large particle size. However, PBN can absorb some of the PS due to its high surface area. On the other hand, FBN can absorb most PS ions from the solution although its surface area is intermediate in comparison to those of CBN and PBN, indicating that NH_2_ positive functional groups on the CBN surface are extremely important for the absorption of PS (S_8_^2−^) ion regardless of its lower surface area [122]. Owing to the positive role of functionalized BNNTs as evidenced in the study by Fan et al., BNNTs should also be considered for battery applications. For battery applications, high concentration and pure electrolytes are mostly used [123]. Therefore, fouling may not be expected to be as high as in the other type of membrane applications. However, it is still a critical issue for battery separator membranes [124]. Hence, bio-fouling, organic fouling, colloidal and scaling resistances of BNNT battery separators should also be investigated in the future studies.

PEMs or proton-electrolyte membranes are semipermeable and electronic insulator membranes that conduct protons. Currently, there is no reported study on this topic. However, the high mechanical and thermal properties of BNNTs make it promising for future applications.

## 5. Conclusions

Since the discovery of BNNT, various studies have already reported its superior properties that can lead to enhanced performance in several applications. Mechanical strength, thermal conductivity, and resistance; constant wide-band-gap insulation, and paintable ultra-pure white color of BNNT already rendered it a widely used material, as well as promising materials for membranes. BNNT-based membranes exhibit high permeation, selectivity, H_2_ adsorption, and cytocompatibility compared to 2D materials such as CNTs. Thus, BNNT membranes are expected to be taking a great role in energy and environmental applications. Regarding this, current research has already shown that BNNTs provide: enhanced performances in osmotic energy conversion, high fluxes and rejections at low pressures for water treatment process, high selectivity and diffusivity in gas separation process, and longer life for batteries by providing stronger and durable membrane separators.

However, most of the current studies still employ MD simulations, and these studies are mainly focusing on the separation properties of BNNT membranes. Therefore, in situ laboratory and pilot-scale studies are required for the more extensive application of BNNTs in membrane science. That is, computer models may not fully consider various external parameters, such as humidity, temperature and microbials in the media. Through laboratory and pilot scale studies, the fouling performance, life duration, resistance to environmental conditions, and the performance loss over time of BNNT membranes should be investigated further. In particular, biofouling resistance loss of BNNT membranes that may be expected to be a result of their electric insulation properties should be critically considered.

## Figures and Tables

**Table 1:** Advantages and disadvantages of BNNT fabrication methods.

Fabrication Method	Advantage	Disadvantage
Annealing of ball-milled BN powders in nitrogen (Ball milling) [24]	Faster growth [24] Longer BNNTs [25] Large quantities can be produced [24]	Usually for bamboo type [24] Difficult to remove B/B–N precursors from the products [9]
BN substitution method from CNT templates [26]	Time efficiency Low cost [26,27]	Low crystallization Low BNNT yield Incomplete substitution of C atoms [27]
Borazine as precursor CVD [17]	High yield Low-pressure requirement Long, concentric, and crystalline nanotubes [17]	Impurity [17]
Boron and magnesium oxide as precursors for CVD (BOCVD) [28]	Rapid production Large quantities can be obtained Less harmful Extremely pure (snow-white) BNNTs [28]	Encapsulated matter near the fracture surface Most nanotubes exhibit an open tip typically with an irregular fracture surface [28]
Heating of Hexagonal BN [29,30]	Effective route for synthesizing SBNNTs High yield Good crystallization Long BNNTs [30]	• High-temperature requirement [29,30]
Laser Ablation/Vaporization [31]	Higher axial growth rate than the radial growth rate [31] Efficient method to produce SBNNTs [31,32]	Presence of byproducts [32] Inefficient to produce MBBNTs [31,32]
Plasma Arc discharge [8]	• Simple and inexpensive setup [33]	Low BNNT yield [9] Impurities [34,35]
Pressure Vapor/Condenser Method (PVC) [36]	No catalyst required Highly crystalline BNNTs Very long and small-diameter BNNTs [36]	• A significant fraction within the yarns of BN phases [9]

**Table 2:** Comparison of BNNT vs. CNT.

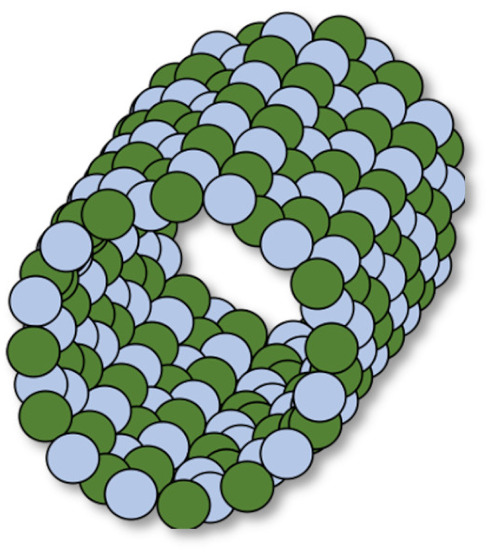		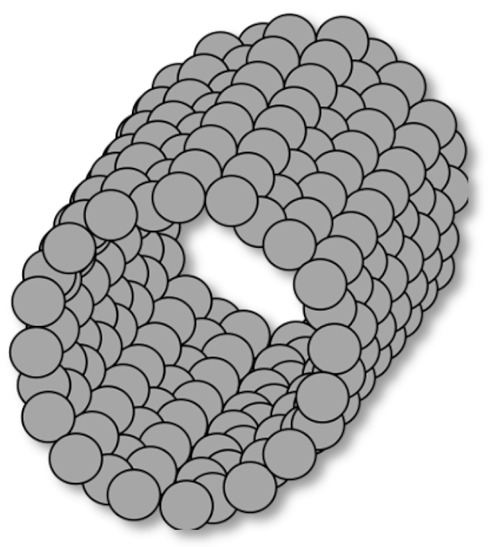	
BNNTs	BNNTs and CNTs		CNTs
✓ White ✓ Covalent bonds with ionic components [15,39,40] ✓ Constant wide-band-gap insulator regardless of chirality [41,42] ✓ Resistant to thermal oxidation [43] ✓ Piezoelectric properties ✓ High hydrogen adsorption [11] ✓ Violet or ultraviolet luminescence [44,45] ✓ Cytocompatible and safe [46] ✓ Neutron radiation shielding [47]	✓ High mechanical strength and stiffness [48,49] ✓ Lightweight nanomaterials ✓ High thermal conductivity and stability [49] ✓ Relatively low affinity to bio-functionalization (non-covalent interactions) [50] ✓ Hydrophobic (non-functionalized) [51]		✓ Black ✓ Covalent bonding [15,39,40] ✓ Narrow band gap (dependent on chirality) semiconductor or metal [15,52] ✓ Not resistant to burning [43] ✓ Infrared luminescence [53] ✓ Potentially toxic [54]
